# Wistar Kyoto Rats Display Anhedonia In Consumption but Retain Some Sensitivity to the Anticipation of Palatable Solutions

**DOI:** 10.3389/fnbeh.2020.00070

**Published:** 2020-06-03

**Authors:** Rebecca L. Wright, Gary Gilmour, Dominic M. Dwyer

**Affiliations:** ^1^School of Psychology, Cardiff University, Cardiff, United Kingdom; ^2^Lilly Research Centre, Eli Lilly & Co. Ltd., Erl Wood Manor, United Kingdom

**Keywords:** WKY, depression, anhedonia, consummatory, anticipatory, contrast

## Abstract

The Wistar Kyoto (WKY) rat has been proposed as a model of depression-like symptoms. However, anhedonia—a reduction in the response to normatively rewarding events—as a central depression symptom has yet to be fully assessed in this model. We compared WKY rats and Wistar controls, with stress-susceptibility examined by applying mild unpredictable stress to a subset of each group. Anhedonia-like behavior was assessed using microstructural analysis of licking behavior, where mean lick cluster size reflects hedonic responses. This was combined with tests of anticipatory contrast, where the consumption of a moderately palatable solution (4% sucrose) is suppressed in anticipation of a more palatable solution (32% sucrose). WKY rats displayed greatly attenuated hedonic reactions to sucrose overall, although their reactions retained some sensitivity to differences in sucrose concentration. They displayed normal reductions in consumption in anticipatory contrast, although the effect of contrast on hedonic reactions was greatly blunted. Mild stress produced overall reductions in sucrose consumption, but this was not exacerbated in WKY rats. Moreover, mild stress did not affect hedonic reactions or the effects of contrast. These results confirm that the WKY substrain expresses a direct behavioral analog of anhedonia, which may have utility for increasing mechanistic understanding of depression symptoms.

## Introduction

Depression is a highly debilitating disorder with symptoms that manifest at the psychological, behavioral and physiological levels. With higher prevalence than other psychiatric disorders, it has been reported that approximately 16% of people will develop depression at some point over their lifetime (Kessler et al., 2003), and is currently a leading global cause of disability (WHO, 2020). While the presentation of depression is varied, a central symptom is an anhedonia (American Psychiatric Association, 2013)—a reduction in the response to normatively rewarding events (Ribot, 1897; Gorwood, 2008), including both consummatory (in the moment pleasure) and anticipatory (expected pleasure) deficits (Gard et al., 2006; Rizvi et al., 2016). Although pharmacotherapy is typically the first-line treatment for depression, current antidepressant drugs are only partially effective (approximately 20% better recovery rates than no-treatment controls), with a slow onset of action (4-6 weeks), and with few genuinely new compounds recently introduced clinically (Belzung, 2014; Willner and Belzung, 2015). Valid animal models play a central role in the investigation of basic pathology and the development of novel therapeutic techniques (Overstreet, 2012; Willner and Belzung, 2015). Much of the animal modeling of depression has focused on the application of stress (e.g., the chronic mild stress procedure), but applying this to genetically “normal” animals does not account for the evidence that there are material differences between individuals in the risk of depression and thus that considering dispositional factors is vital in a truly valid modeling approach (Willner and Belzung, 2015; Wang et al., 2017). A variety of rodent modes of susceptibility to depression, stress, and/or anxiety have been proposed, amongst these the Wistar-Kyoto (WKY) rat is becoming increasingly recognized for its promise in reflecting key aspects of depression (Overstreet, 2012; Nam et al., 2014; Willner and Belzung, 2015; Wang et al., 2017).

Originally bred as controls for the Spontaneously Hypertensive Rat (Louis and Howes, 1990), WKY animals were soon noted as having high susceptibility to stress-ulceration (e.g., Paré, 1989b; Paré and Redei, 1993; Paré and Kluczynski, 1997), and subsequent research revealed greater expression of behavioral markers in rodent depression studies (e.g., Paré, 1989a, b; Paré and Redei, 1993; Paré and Kluczynski, 1997; Rittenhouse et al., 2002), as well as deficits on other tests relating to anxiety and/or depression (e.g., Paré, 1994; Solberg et al., 2001; Pardon et al., 2002; De La Garza and Mahoney, 2004; Ferguson and Gray, 2005; Shepard and Myers, 2008; D’Souza and Sadananda, 2017). Moreover, the WKY rat appears to be insensitive to some common antidepressant compounds (e.g., Lahmame et al., 1997; López-Rubalcava and Lucki, 2000; Tejani-Butt et al., 2003), suggesting it may be particularly suitable as a model of treatment-resistant depression. Despite this promising literature, the key question of whether WKY rats truly display anhedonia remains to be answered: partially because of some inconsistencies between reported results and because of the nature of the testing methods used.

In assessing anhedonia, traditional tests have relied on the consumption of (or preference for) sweetened solutions such as sucrose or saccharin, on the assumption that a lowered hedonic response to palatable sweet flavors would be directly analogous to anhedonia (Willner et al., 1987; Papp et al., 1991; Muscat and Willner, 1992; Forbes et al., 1996). But while palatability certainly can influence consumption, a great many other factors will also produce consumption changes, including differences in motivation and physiological need, post ingestive effects, and satiety (Booth et al., 1972; Warwick and Weingarten, 1996; Brennan et al., 2001; Dwyer, 2012; Lewis et al., 2019). Thus, reductions in the consumption or preference for sweet flavors are not uniquely consistent with anhedonia and assuming consumption changes reflect hedonic responses is problematic. Moreover, direct intake measures only address the consummatory aspects of anhedonia and not anticipatory anhedonia. Also, although there are several reports that WKY rats display deficits in sweet flavor consumption or preference (e.g., Malkesman et al., 2005; Luo et al., 2015; Burke et al., 2016; Shoval et al., 2016; D’Souza and Sadananda, 2017; Fragale et al., 2017) others report no such deficits and even enhancements of consumption/preference (e.g., Nam et al., 2014; Mileva and Bielajew, 2015).

In response to the fact that consumption-only measures do not directly assess hedonic reactions, more sensitive assays of hedonic behavior have been developed. For example, orofacial reactivity tests (Grill and Norgren, 1978) use the fact that responses to intra-orally infused solutions can be separated into appetitive and aversive behavior patterns. This measure has been extensively used in the context of separating hedonic and other components of reward processing and their biological basis (e.g., Berridge, 1996; Berridge and Robinson, 1998; Castro and Berridge, 2014). While they have been used in a highly productive manner, orofacial reactivity tests have some practical limitations, particularly concerning the effective life of implanted oral cannula and the separation of voluntary consumption from the reactions to infused solutions. An alternative approach to assessing hedonic responses relies on the analysis of the microstructure of voluntary consumption—rodents typically produce clusters of licks separated by pauses, and the mean number of licks per cluster displays a positive monotonic relationship with the concentration of palatable sucrose solution (e.g., Davis and Smith, 1992; Davis and Perez, 1993; Spector et al., 1998), a negative relationship with an unpalatable solution such as quinine (Hsiao and Fan, 1993; Spector and St John, 1998), as well as being sensitive to pharmacological interventions known to affect palatability in humans (Asin et al., 1992; Higgs and Cooper, 1998). Critically, lick cluster size is not simply a proxy for consumption: although cluster size increases with increased sucrose concentration, the amount consumed decreases at high concentrations due to satiety (e.g., Ernits and Corbit, 1973); while studies of conditioned taste aversion and preference have also shown that palatability and consumption can dissociate (e.g., Dwyer et al., 2008, 2009, 2012, 2013; Dwyer, 2009). Thus, in the present experiments, we used the analysis of lick cluster size to provide a means of selectively assessing palatability responses. We have previously used this method to demonstrate the presence of an anhedonic profile in animals subject to social- (Dwyer, 2012) or handling-stress (Clarkson et al., 2018), as well as in a genetic model for Silver Russell Syndrome (McNamara et al., 2016) while ruling out a hedonic disturbance in a model for Prader-Willi syndrome (Davies et al., 2015) and NMDA antagonist models for psychosis (Lydall et al., 2010).

As previously mentioned, anhedonia in depression can separate between consummatory and anticipatory deficits (Gard et al., 2006; Rizvi et al., 2016). Moreover, there is evidence from patient studies that both consummatory and anticipatory hedonic deficits are present in depression (McFarland and Klein, 2009; Liu et al., 2011). To address both anticipatory and consummatory aspects of anhedonia in WKY rats we used an anticipatory contrast procedure (e.g., Flaherty and Rowan, 1985; Flaherty, 1996). This involves giving rats access to two solutions each day when the first solution is of a lower concentration than the second (e.g., 4% then 32% sucrose), both consumption of, and the lick cluster size for, the initial solution is suppressed compared to when the two solutions are of equal concentration (Arthurs et al., 2012; Wright et al., 2013). Thus, anticipatory contrast involves the downregulation of the current hedonic experience based on the expectation of a future event of high value and reflects anticipatory aspects of hedonic responses. We complemented this anticipatory test with the analysis of licking microstructure during the simple consumption of a range of sucrose concentrations to examine consummatory hedonic responses.

Also, it is uncertain whether the deficits seen with WKY rats are exacerbated by external stressors (compare Paré and Kluczynski, 1997; Nam et al., 2014 with Malkesman et al., 2006; Sterley et al., 2011). Thus we used a factorial design whereby both WKY and Wistar control groups were divided and an attenuated chronic mild stress procedure (Willner et al., 1987, 1992) was applied to half of the rats in each group. The stressor involved exposures to a brief swimming event, and thus also provided an opportunity to confirm that the typical WKY deficit on this task was present in the current cohort of animals. Importantly, the frequency of the stressor application was reduced compared to the typical chronic mild stress procedure (3 per week as opposed to daily treatments) to test whether WKY animals would be susceptible to a lower level of stress than that required to produce effects in control animals.

## Materials and Methods

### Animals

Male Wistar (*N* = 24) and Wistar Kyoto (WKY, *N* = 24) rats were used. Both were from Charles River (UK) breeding stocks and were delivered to Cardiff University at approximately 11 weeks of age. On arrival, both Wistar and WKY rats were split into two weight-matched groups of twelve into either a “No-stress” or a “Stress” condition [Mean Weights (±SEM): Wistar No-Stress 177.8 g (±3.9); Wistar Stress 182 g (±6.8); WKY No-stress 182.3 g (±4.2); WKY Stress 178.4 g (±7.9)]. No-stress rats were housed in pairs and their home cages included standard environmental enrichment (tubes and gnawing sticks). Stress rats were singly housed in a separate room and no environmental enrichment was provided. Before the start of experimental work, all animals were placed on a food-restricted diet, which maintained them between 85 to 95% of their free-feeding weights (this was matched to the expected growth rate of free-feeding animals, and thus weights during the experimental periods exceeded the original free-feeding weights). Their food ration was given in their home cage approximately 30 min after behavioral procedures (or around 5 pm if there were no procedures on that day). Careful monitoring was employed throughout to ensure that rat weights, as a percentage of free-feeding weights, did not differ significantly between the two strain and stress conditions. Animal weight data during the experimental periods and its analysis can be found in Supplementary Table S3 (for the Anticipatory Contrast study) and Supplementary Table S4 (for the Consumption study). Food restriction was performed to motivate the consumption of the caloric sugar solutions and also allowed for the motivational state of the stressed and non-stressed animals to be matched in case the stress procedures created a difference in energy demands between conditions. While food restriction may affect lick cluster size (compare Davis and Perez, 1993 with Spector et al., 1998) this is unlikely to have a material effect on the results obtained here because the food restriction was applied to all animals and groups were equivalent in terms of the effects of restriction on body weight as a percentage of free-feeding weights (see Supplementary Tables S3, S4). Unless otherwise specified, rats were held under a 12-h light/dark cycle. Experimental sessions were performed during the light phase, beginning at approximately 11 am, and were conducted 6 to 7 days per week. Due to the provision of food rations and application of stress procedures (or handling in the No-stress conditions) during the light phase, rats had 2 weeks to adapt to procedures occurring in this phase, thus minimizing the impact of testing normally nocturnal animals during the day. This project was considered and approved by the Cardiff University Animal Welfare and Ethical Review Board (AWERB) and all experiments were conducted following the United Kingdom Animals Scientific Procedures Act, 1986.

### Mild Stressor Tests

Rats in the Stress condition underwent a series of mild social and environmental stressors which commenced a week before testing. This continued throughout these experiments. Each week, rats in the stress group were exposed to three of five possible stressors: wet bedding, overnight illumination, cage swap with an unfamiliar rat, pair-up with an unfamiliar rat, and a brief swim test. Details of the stress procedures, including the relationship to other experimental manipulations, are shown in the Supplementary Materials (see Supplementary Tables S1, S2). The identity of the stressor was randomly allocated, as was the day on which it was given. When stress manipulations were to occur on the same day as an experimental session, the stressor was applied after the training or test session had been carried out. Rats in the No-stress condition were gently handled on the same days as stress procedures were applied.

During brief swim tests, the rats’ behavior was recorded *via* a camcorder mounted above the water cylinders. Data were scored using a time sampling technique, whereby the rats’ predominant behavior was noted every 2 s across the 120 s test. Recording commenced as soon as the rats had entered the water. Their behavior was scored as either “Active,” “Escape” or “Immobile.” Active behavior was recorded when the rat was swimming, climbing or diving. Thus, rats would be considered “active” if they made upward-directed movements of the forepaws, horizontal movements across the cylinder (including rapid changes in the rat’s direction) or dived to the bottom of the cylinder before resurfacing. Immobile behavior was recorded if rats were floating in the water without any signs of struggling. Small movements of the back limbs were permitted in this category if they served only to keep the animals head out of the water. Escape behavior was recorded if the rat was able to leave the cylinder. This would be considered as one escape. For every subsequent 2 s period where the rat was out of the water, an “X” would be recorded so that it was not included in subsequent analysis. The percentage of time spent active, immobile or escaping was then calculated for each animal.

As the primary observer was not blind to rat strain, a single session, chosen at random, was re-scored by a secondary observer (who was blind to the strain) using the criterion outlined above. Inter-rater reliability assessment revealed a strong positive correlation between the two observers’ immobility scores, *r*_(22)_ = 0.975, *p* < 0.001.

### Negative Anticipatory Contrast

#### Apparatus

Testing was conducted in six automated drinking chambers (Med Associates Inc., St Albans, VT, USA), measuring 30 × 24 × 21 cm, and comprised of two clear Perspex and two aluminum walls. The chamber floor consisted of 19 steel rods, 4.8 mm in diameter and 16 mm apart. Approximately 5 cm above the grid floor, two holes each of 1 cm diameter were positioned on each side of one aluminum wall to allow the rat access to the solutions. Solutions were delivered through the right and left access holes by 50 ml cylinders with ball-bearing metal drinking-spouts. These were mounted to the cage *via* motorized holders that held the spout flush with the outside of the chamber and retracted it as required. Contact sensitive lickometers registered the timing of each lick made by the animal to the nearest 0.01 s, and a computer running MED-PC software controlled the equipment and recorded the data. The solutions used were 4% and 32% (wt/wt) sucrose formulated using commercial-grade cane sugar and deionized water.

#### Procedure

All rats were habituated to the drinking boxes for 10 min each day for 3 days before the pre-training phase of the experiment. This was to overcome stress effects caused by a novel environment that may have differentially affected the potentially stress-sensitive WKY rats. No solutions were made available during this habituation. During pre-training, rats were water restricted for 22 h and then given access to water for 10 min from both the left- and right-hand side of the drinking chamber. Only one pre-training day was given, after which rats were returned to *ad libitum* water and remained so for the duration of the experiment. During initial training, drinking spouts were positioned inside the chamber to allow for easy detection by the rats, spouts were gradually moved back to be flush with the outside of the chamber across the first 3 days of training.

On each subsequent training day, the solution pairings were manipulated within subjects. Rats were presented with either a 4% sucrose solution followed by more 4% sucrose (the 4–4 condition) or a 4% sucrose solution followed by a 32% sucrose solution (the 4–32 condition). These daily solution pairings were presented in double alternation (e.g., ABBAABBA) and different contextual cues were used to signal which of the two solution pairings was in operation each day. There were thus 32 total testing days, with 16 days in each of the 4–4 and 4–32 conditions. For half the animals, context 1 (consisting of bright light and normal grid floor) was paired with the 4–4 condition, and context 2 (consisting of dim light provided by a table lamp and a wire mesh floor insert) was paired with the 4–32 condition. The remaining subjects had the opposite pairings. The first solution in the pair was made available for 3 min on the left-hand side of the chamber. Following a 4-s inter-solution interval, the second solution was then made available for 6 min on the right-hand side of the chamber. The apparatus and procedures are the same we have reported previously (Wright et al., 2013).

#### Consumption and Lick Cluster Size Analysis

Consumption was assessed by weighing the bottle before and after each experimental run. Lick cluster size (defined as the mean number of licks per cluster) was extracted from the MED-PC data. As in our lab’s previous experiments using these general methods and equipment (e.g., Lydall et al., 2010; Dwyer et al., 2011, 2018; Wright et al., 2013), a cluster was defined as series of licks, with each lick separated by no more than a 0.5 s interval. The same criterion had been adopted by Davis and his colleagues (e.g., Davis, 1989; Davis and Smith, 1992; Davis and Perez, 1993). Drinking data were collated into 2-session blocks.

### Sucrose Consumption

The same animals were re-tested to examine sucrose consumption across a range of concentrations without contrast. The solutions used were 2, 8 and 24% (wt/wt) sucrose made daily with deionized water and the apparatus was as described previously. Because rats had already undergone anticipatory contrast testing involving multiple drinking sessions no pre-training or habituation was necessary.

Rats were given access to one of the three sucrose concentrations which were always made available from the left-hand side of the drinking chamber. Each concentration was given for three consecutive days and the order of sucrose presentations was counterbalanced so that half of the rats received the sucrose in order of increasing concentration (2–8–24) and the other half received them in order of decreasing (24–8–2) concentration. A two-day rest was given before the next concentration in the sequence was presented. All solutions were made available for 10 min each day.

Consumption and lick cluster size analyses were conducted using the same parameters described for anticipatory contrast. To minimize any effects of transition between concentration, data were analyzed across the last 2 days of exposure for each solution concentration. One animal (a WKY No-stress rat) was excluded from the descriptive and inferential statistics reported for the sucrose consumption phase. This was due to abnormally high lick cluster size displayed by this animal for 32% sucrose, more than 3.5 standard deviations above the group mean^1^.

### Data Analysis

Immobility data in the brief swim test was analyzed with mixed ANOVA with a within-subject factor of the sessions (1–5), and a between-subject factor of strain (Wistar vs. WKY). Data from anticipatory contrast and consumption phases were analyzed with mixed ANOVAs with between-subject factors of strain (Wistar vs. WKY) and stress (Stress vs. No stress), plus within-subject factors appropriate for each experiment: For the anticipatory contest, there were within-subject factors of the block (1–8) and contrast condition (4–4 or control condition vs. 4–32 or contrast condition); for consumption, there was a within-subject factor of concentration (2, 8, 24%). An alpha level of 0.05 was adopted as the level of significance throughout and Greenhouse-Geisser corrections for violations of the assumption of sphericity applied as appropriate.

## Results

### Stress Manipulation and Brief Swim

**Figure 1:**
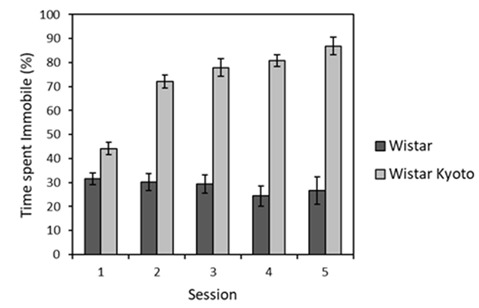
Mean percentage of time immobile (±SEM) during 2 min brief swim sessions for animals subjected to mild stressor protocols (Wistar: dark bars; WKY: light bars). WKY rats were significantly more immobile than Wistar rats, and the levels of immobility significantly increased across sessions in WKY but not Wistar rats (see “Stress Manipulation and Brief Swim” section, for details). See Supplementary Table S2 for the timing of these sessions relative to the other experimental procedures.

Repeated swim test exposures occurring throughout the stressor protocol were analyzed over time (see Supplementary Table S2 for the relationship between brief swim tests and other stressor events). It was found that WKY rats spent more time immobile compared to their Wistar counterparts across all sessions (Figure 1). Time spent immobile also generally increased across sessions for WKY animals, unlike for the Wistar strain. ANOVA yielded main effects of session (*F*_(4,88)_ = 10.8, *p* < 0.001, $\eta_{\text{p}}^{2}$ = 0.33) and strain (*F*_(1,22)_ = 187.7, *p* < 0.001, *η*^2^ = 0.90), as well as a session × strain interaction (*F*_(4,88)_ = 14.5, *p* < 0.001, $\eta_{\text{p}}^{2}$ = 0.45). Further inspection of the interaction revealed that that immobility significantly increased across sessions for WKY rats (*F*_(4,44)_ = 36.9, *p* < 0.001, *η*^2^ = 0.77) but not Wistar rats (*F* < 1). Thus, the typical pattern of enhanced immobility for WKY animals in swim tests was replicated here.

### Negative Anticipatory Contrast

#### First Solution Consumption—Anticipatory Contrast

**Figure 2:**
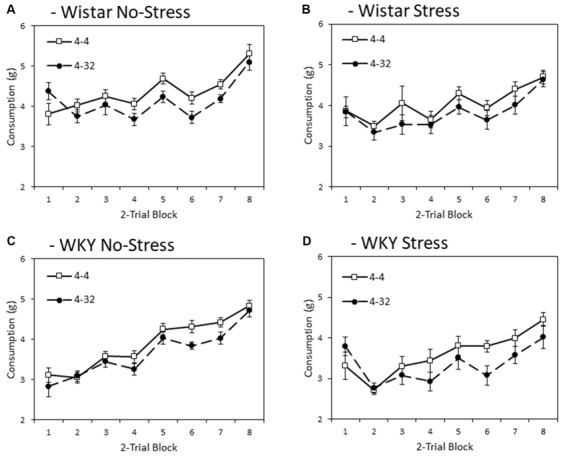
Anticipatory contrast in mean consumption (±SEM) of the initially presented 4% sucrose solution as a function of whether it was followed by either 4% sucrose (open symbols) or 32% sucrose (filled symbols), separated by strain (Wistar: Panels **A,B**; WKY: Panels **C,D**) and stress groups (No-Stress: Panels **A,C**; Stress: Panels **B,D**). The data presented is averaged over two-day blocks. The solution was available for 3 min per day. WKY rats consumed significantly less than Wistar rats, and stressed rats consumed significantly less than no-stress rats, and these effects did not interact. Critically, there was an anticipatory contrast effect (consumption of 4% sucrose was significantly lower when followed by 32% sucrose than 4% sucrose) and this effect did not interact with strain or stress manipulations (see “First Solution Consumption—Anticipatory Contrast” section for details).

Figure 2 depicts consumption of the initially presented 4% sucrose solution across training blocks. In general, WKY rats consumed significantly less than Wistars did (main effect of strain: *F*_(1,44)_ = 13.8, *p* < 0.001, $\eta_{\text{p}}^{2}$ = 0.24), and stressed rats consumed less than non-stressed rats (main effect of stress: *F*_(1,44)_ = 6.1, *p* = 0.018, $\eta_{\text{p}}^{2}$ = 0.12), although no significant interaction was found between these factors (strain × stress: *F* < 1). These effects significantly varied across training blocks (main effect of block: *F*_(4.6,199.0)_ = 59.9, *p* < 0.001, $\eta_{\text{p}}^{2}$ = 0.58), with strain and stressor effects increasing in effect size across blocks (block × strain: *F*_(4.6,199.0)_ = 3.4, *p* = 0.008, $\eta_{\text{p}}^{2}$ = 0.07; block × stress: *F*_(4.6,199.0)_ = 3.2, *p* = 0.010, $\eta_{\text{p}}^{2}$ = 0.07); block × strain × stress: *F*_(4.6, 199.0)_ = 2.6, *p* = 0.029, $\eta_{\text{p}}^{2}$ = 0.06).

Importantly, a significant anticipatory contrast effect was evident (main effect of contrast: *F*_(1,44)_ = 28.4, *p* < 0.001, $\eta_{\text{p}}^{2}$ = 0.39) where consumption of the initially presented 4% sucrose solution was lower when followed by the more palatable 32% sucrose compared to when followed by a second presentation of 4% sucrose. Critically, this anticipatory contrast effect did not differ significantly between strains and did not significantly interact with stress (contrast × strain, contrast × stress, contrast × strain × stress interactions: *F*s < 1). Consistent with anticipatory contrast effects emerging as a function of learning over training blocks, a significant block × contrast interaction was found (*F*_(4.0,175.8)_ = 4.0, *p* = 0.004, $\eta_{\text{p}}^{2}$ = 0.08). This learning function did not significantly interact with strain or stress (block × contrast × strain, block × contrast × stress (*F*s < 1), block × contrast × strain × stress (*F*_(4.0,175.8)_ = 2.0, *p* = 0.097, $\eta_{\text{p}}^{2}$ = 0.04)^2^.

#### First Solution Lick Cluster Size—Anticipatory Contrast

**Figure 3:**
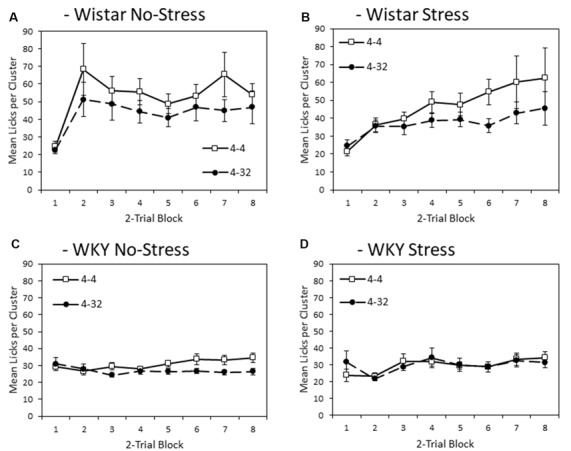
Anticipatory contrast in mean lick cluster size (LCS: ±SEM) of the initially presented 4% sucrose solution as a function of whether it was followed by either 4% sucrose (open symbols) or 32% sucrose (filled symbols), separated by strain (Wistar: Panels **A,B**; WKY: Panels **C,D**) and stress groups (No-Stress: Panels **A,C**; Stress: Panels **B,D**). The data presented is averaged over two-day blocks. The solution was available for 3 min per day. WKY rats displayed a significantly lower mean lick cluster size than Wistar rats, but there was no effect of stress or stress × strain interaction. Critically, there was an anticipatory contrast effect (lick cluster size, indicating hedonic responses, for 4% sucrose was significantly lower when followed by 32% sucrose than 4% sucrose) and this effect was significantly reduced in WKY compared to Wistar rats (see “First Solution Lick Cluster Size—Anticipatory Contrast” section for details). Differential lick cluster sizes for the 4% solution depending on the subsequent concentration of sucrose solution offers a potential measure of anticipatory anhedonia.

Figure 3 depicts mean lick cluster size (LCS) during consumption of the initially presented 4% sucrose solution across training blocks. Overall, WKY rats produced fewer licks per cluster compared to Wistar rats (main effect of strain (*F*_(1,44)_ = 17.2, *p* < 0.001, $\eta_{\text{p}}^{2}$ = 0.28). Stress did not significantly affect lick cluster size (Main effect of stress; stress × strain interaction, *F*s < 1). As with consumption, significant anticipatory contrast effects were evident for lick cluster size (Main effect of contrast: *F*_(1,44)_ = 9.4, *p* = 0.004, $\eta_{\text{p}}^{2}$ = 0.18), with lick cluster size for the initially presented 4% sucrose solution lower when followed by the more palatable 32% sucrose compared to when followed by a second presentation of 4% solution. Critically, this contrast effect was significantly smaller in WKY than Wistar rats (contrast × strain: *F*_(1,44)_ = 4.4, *p* = 0.041, $\eta_{\text{p}}^{2}$ = 0.09). The contrast effect was not influenced by stress (contrast × stress, contrast × strain × stress, *F*s < 1). Consistent with the emergence of effects over training, there was a main effect of block (*F*_(3.6,156.4)_ = 7.5, *p* < 0.001, $\eta_{\text{p}}^{2}$ = 0.15), and a block × contrast interaction (*F*_(4.0,175.8)_ = 3.0, *p* = 0.020, $\eta_{\text{p}}^{2}$ = 0.06). There was also a block × strain interaction (*F*_(3.6,156.4)_ = 6.1, *p* < 0.001, $\eta_{\text{p}}^{2}$ = 0.12), but not block × stress (*F*_(3.6,156.4)_ = 2.1, *p* = 0.091, $\eta_{\text{p}}^{2}$ = 0.05), nor block × strain × stress (*F*_(3.6,156.4)_ = 1.264, *p* = 0.228, $\eta_{\text{p}}^{2}$ = 0.03). There were no significant interactions between block × contrast × strain, block × contrast × stress (*F*s < 1), or block × contrast × strain × stress (*F*_(4.0,175.8)_ = 1.4, *p* = 0.194, $\eta_{\text{p}}^{2}$ = 0.03).

To aid interpretation, the critical contrast × strain interaction was explored further with separate ANOVAs conducted on each strain with factors of block and contrast condition. Wistar rats showed significant main effects of contrast (*F*_(1,23)_ = 7.5, *p* = 0.012, $\eta_{\text{p}}^{2}$ = 0.25) and block (*F*_(3.1,72.0)_ = 7.6, *p* < 0.001, $\eta_{\text{p}}^{2}$ = 0.25), but no contrast × block interaction (*F*_(3.4,79.3)_ = 1.7, *p* = 0.122, $\eta_{\text{p}}^{2}$ = 0.07). WKY rats presented with a different pattern of findings, where there was no significant main effect of contrast (*F*_(1,23)_ = 2.9, *p* = 0.103, $\eta_{\text{p}}^{2}$ = 0.11) or block (*F*_(2.8,63.7)_ = 1.776, *p* = 0.165, $\eta_{\text{p}}^{2}$ = 0.07), but there was a significant contrast × block interaction (*F*_(3.8,88.1)_ = 2.9, *p* = 0.008, $\eta_{\text{p}}^{2}$ = 0.11). These results suggest that lick cluster size in WKY rats did show some sensitivity to contrast as training progressed, but far less than the Wistar controls, possibly due to the generally low levels of hedonic response seen in the WKY animals overall. While the anticipatory contrast effect was attenuated for WKY No-stress and Stress rats with a lower lick cluster size difference for first presentation 4% sucrose solution between the two contrast conditions than for the Wistar controls, a contrast effect in WKY animals was apparent by the end of the experiment, most obviously for the No-stress group.

#### Second Solution Consumption—Low vs. High Sucrose Concentration

**Figure 4:**
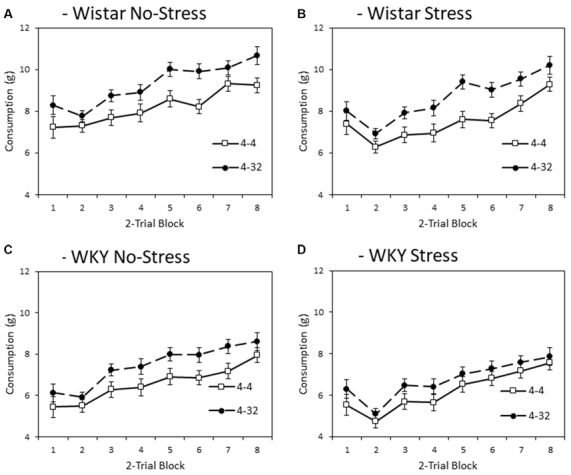
Mean consumption (±SEM) of the second presentation solution made available each day as a factor of strain (Wistar: Panels **A,B**; WKY: Panels **C,D**) and stress (No-Stress: Panels **A,C** ; Stress: Panels **B,D**). Open symbols represent the consumption of 4% sucrose in the second bottle and filled symbols represent the consumption of 32% sucrose in the second bottle. Data presented is averaged across 2-session blocks. Solutions in the second bottle were made available for 6 min. WKY rats consumed significantly less than Wistar rats, and stressed rats consumed significantly less than no-stress rats, but these effects did not interact. Also, consumption of 32% sucrose was higher than that of 4% sucrose, but this difference was not significantly influenced by strain or stress (see “Second Solution Consumption—Low vs. High Sucrose Concentration” section for details).

Figure 4 depicts consumption of second presentation solution (4% or 32% sucrose) across training blocks. Note that consumption levels were larger than for first presentation because the first bottle was only made available for 3 min and the second bottle for 6 min. Like the first presentation data, WKY rats generally consumed less during second presentation compared to Wistars (main effect of strain: *F*_(1,44)_ = 65.2, *p* < 0.001, $\eta_{\text{p}}^{2}$ = 0.60), and stressed animals consumed less than non-stressed animals (main effect of stress: *F*_(1,44)_ = 8.1, *p* = 0.007, $\eta_{\text{p}}^{2}$ = 0.16), again in the absence of a significant stain × stress interaction (*F* < 1). In general, there was higher consumption of the more palatable 32% sucrose solution compared with the moderately palatable 4% sucrose solution (main effect of solution concentration: *F*_(1,44)_ = 23.8, *p* < 0.001, $\eta_{\text{p}}^{2}$ = 0.35). The differential consumption of the 4% and 32% sucrose solutions was not significantly impacted by factors of strain or stress (concentration × strain: *F*_(1,44)_ = 1.1, *p* = 0.294, $\eta_{\text{p}}^{2}$ = 0.03, concentration × stress, concentration × strain × stress, *F*s < 1). Consistent with the progressive increase in consumption as a function of training, there was a main effect of block (*F*_(3.3,145.5)_ = 91.8, *p* < 0.001, $\eta_{\text{p}}^{2}$ = 0.68), which was not significantly impacted by factors of strain, stress or solution concentration (block × strain: *F*_(3.3,145.5)_ = 1.0, *p* = 0.385, $\eta_{\text{p}}^{2}$ = 0.02; block × stress: *F*_(3.3,145.5)_ = 2.5, *p* = 0.058, $\eta_{\text{p}}^{2}$ = 0.05; block × strain × stress, *F* < 1; block × concentration: *F*_(2.8,123.2)_ = 1.689, *p* = 0.176, $\eta_{\text{p}}^{2}$ = 0.04; block × concentration × strain; block × concentration × stress; or block × concentration × strain × stress, *F*s < 1)^3^.

#### Second Solution Lick Cluster Size—Low vs. High Sucrose Concentration

**Figure 5:**
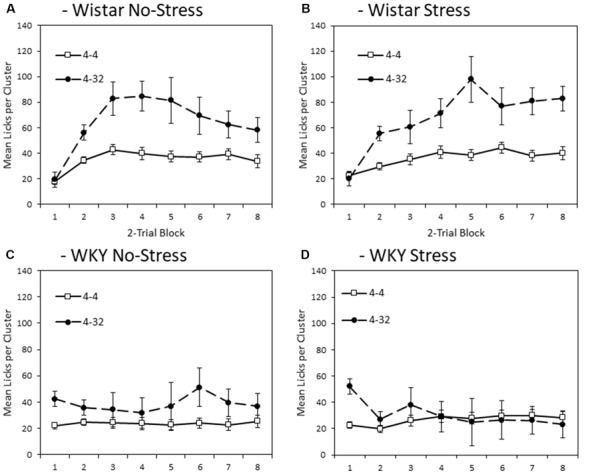
Mean lick cluster size (LCS: ±SEM) for the second solution available each day (4% vs. 32% sucrose) separated by strain (Wistar: Panels **A,B**; WKY: Panels **C,D**) and stress groups (No-Stress: Panels **A,C**; Stress: Panels **B,D**). Open symbols represent responses to the 4% sucrose solution, the filled symbols represent responses to the 32% sucrose solution made available in the second bottle. Data presented is averaged across 2-session blocks. While both WKY and Wistar rats showed significantly higher lick cluster size for 32% than 4% sucrose (reflecting its higher palatability), this difference was significantly smaller in WKY rats and mean lick cluster size overall was significantly lower in WKY rats, indicating a blunted hedonic response (see “Second Solution Lick Cluster Size—Low vs. High Sucrose Concentration” section for details).

Figure 5 depicts mean lick cluster size (LCS) during consumption of second presentation solution (4% or 32% sucrose) across training blocks. Overall, WKY rats exerted significantly fewer licks per cluster compared with Wistar animals (main effect of strain: *F*_(1,44)_ = 18.6, *p* < 0.001, $\eta_{\text{p}}^{2}$ = 0.30). Lick cluster size was not significantly influenced by the application of an external stressor (main effect of stress; stress × strain interaction, *F*s < 1). As for consumption, lick cluster size was significantly greater for 32% sucrose solution compared to 4% sucrose (main effect of concentration (*F*_(1,44)_ = 9.4, *p* < 0.001, $\eta_{\text{p}}^{2}$ = 0.32). Critically, this effect significantly varied with strain (concentration × strain interaction (*F*_(1,44)_ = 5.817, *p* = 0.020, $\eta_{\text{p}}^{2}$ = 0.12), but not with stress (concentration × stress, or concentration × strain × stress interactions (*F*s < 1). In addition, lick cluster size for the second presented solution was also subject to significant change over training (main effect of block: *F*_(3.4,149.9)_ = 7.8, *p* < 0.001, $\eta_{\text{p}}^{2}$ = 0.15). While there was no significant block × concentration interaction (*F*_(3.5,153.5)_ = 1.2, *p* = 0.326, $\eta_{\text{p}}^{2}$ = 0.03), there were both significant block × strain (*F*_(3.4,149.9)_ = 12.1, *p* < 0.001, $\eta_{\text{p}}^{2}$ = 0.22), and block × concentration × strain (*F*_(3.5,153.5)_ = 5.6, *p* < 0.001, $\eta_{\text{p}}^{2}$ = 0.11) interactions. The factor of stress did not significantly interact with any statistical comparison involving the factor of block (block × stress, *F* < 1; block × strain × stress: *F*_(3.4, 149.9)_ = 2.2, *p* = 0.082, $\eta_{\text{p}}^{2}$ = 0.05; block × concentration × stress, *F* < 1; block × concentration × strain × stress: *F*_(3.5,153.5)_ = 1.8, *p* = 0.150, $\eta_{\text{p}}^{2}$ = 0.04).

Further exploration of the critical concentration × strain interaction described above revealed that, although the difference in lick cluster size between 4% and 32% sucrose was smaller in WKY than Wistar rats, it was nevertheless significant in both groups (WKY, main effect of concentration: *F*_(1,23)_ = 4.7, *p* = 0.040, $\eta_{\text{p}}^{2}$ = 0.17; Wistar, main effect of concentration: *F*_(1,23)_ = 16.9, *p* < 0.001, $\eta_{\text{p}}^{2}$ = 0.42). In summary, for Wistar rats, irrespective of stress, lick cluster sizes elicited during consumption of the 32% solution were higher compared with when the 4% solution was consumed, reflecting the greater palatability of this solution. Likewise for WKY rats, lick cluster size was also marginally higher for the 32% sucrose solution than for the 4% sucrose solution, at least early in training. This suggests that, while the hedonic reaction of WKY rats to palatable sucrose is materially blunted, it is not entirely absent.

### Sucrose Consumption

#### Consumption of 2, 8, and 32% Sucrose

**Figure 6:**
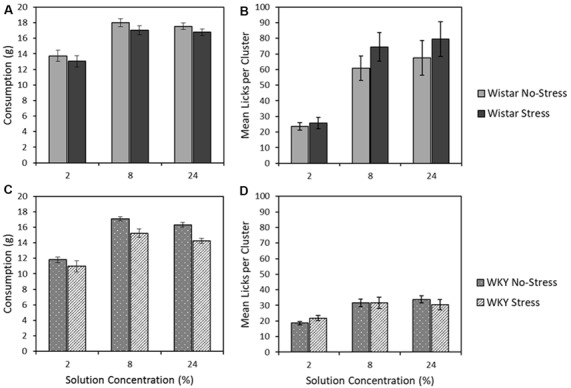
Mean consumption (±SEM]; panels **A,C**) and mean lick cluster size [LCS: panels **B,D** (±SEM) as a function of concentration. This test involves single solution presentations only and thereby removes the anticipatory element of the previous contrast studies. Wistar rats are shown in the upper panels **(A,B)**, WKY rats in the lower panels **(C,D)**. For consumption, WKY rats drank significantly less than Wistar rats, and stressed rats consumed significantly less than no-stress rats, but these factors did not interact (see “Consumption of 2, 8, and 32% Sucrose” section for details). While both WKY and Wistar rats displayed increasing lick cluster size with concentration increases (reflecting the higher palatability of more concentrated solutions), these effects were significantly smaller in WKY rats, which also displayed lower lick cluster size overall: a pattern of effects that is consistent with a partially preserved but extremely blunted hedonic responses in WKY rats (see “Lick Cluster Size for 2, 8, and 32% Sucrose” section for details).

Figures 6A,C depict the mean consumption of the three sucrose concentrations (2, 8 and 24%) for Wistar and WKY rats, separated into Stress and No-stress groups. Sucrose concentration influenced total consumption, with the moderate (8%) solution instead eliciting the highest intake across animals (main effect of concentration: *F*_(1.6,67.6)_ = 194.2, *p* < 0.001, $\eta_{\text{p}}^{2}$ = 0.82). Regardless of concentration, WKY rats consumed significantly less than their Wistar counterparts (main effect of strain: *F*_(1,43)_ = 15.2, *p* < 0.001, $\eta_{\text{p}}^{2}$ = 0.26) and stressed rats consumed significantly less than non-stressed rats (main effect of stress: *F*_(1,43)_ = 7.0931, *p* = 0.011, $\eta_{\text{p}}^{2}$ = 0.14). There was no significant strain × stress interaction (*F* < 1), indicating that stress did not differentially affect intake levels across the Wistar and WKY strains. There was also no strain × concentration (*F* < 1), stress × concentration (*F*_(1.6,67.6)_ = 1.1, *p* = 0.307 $\eta_{\text{p}}^{2}$ = 0.03), or strain × stress × concentration interactions (*F* < 1)^4^.

#### Lick Cluster Size for 2, 8, and 32% Sucrose

Figures 6B,D depict the mean lick cluster size (LCS) elicited by Wistar and WKY rats, separated into the two stress conditions, when consuming each of the three sucrose concentrations. While overall there was a significant concentration-dependent increase in lick cluster size (main effect of solution concentration: *F*_(1.5,64.4)_ = 49.9, *p* < 0.001, $\eta_{\text{p}}^{2}$ = 0.54), Wistar rats in general displayed significantly greater lick cluster size than WKY rats (main effect of strain: *F*_(1,43)_ = 30.7, *p* < 0.001, $\eta_{\text{p}}^{2}$ = 0.42) indicating a deficit in hedonic reactions to sucrose. In addition, a significant strain × concentration interaction was evident (*F*_(1.5,64.3784)_ = 17.9, *p* < 0.001, $\eta_{\text{p}}^{2}$ = 0.29), but stress did not significantly influence effects of other factors on lick cluster size (main effect of stress; strain × stress interaction; stress × concentration; concentration × strain × stress interactions, *F*s < 1).

Further exploration of the critical concentration × strain interaction was explored by performing separate ANOVA analyses for WKY and Wistar rats. In each analysis rats, a significant main effect of concentration (Wistar: *F*_(2,44)_ = 36.0, *p* < 0.001, $\eta_{\text{p}}^{2}$ = 0.62; WKY: *F*_(2,42)_ = 26.9, *p* < 0.001, $\eta_{\text{p}}^{2}$ = 0.56) was found, while there was no main effect of stress (Wistar: *F*_(1,22)_ = 1.0, *p* = 0.486, $\eta_{\text{p}}^{2}$ = 0.02; WKY: *F* < 1), and no stress × concentration interaction (Wistar: *F* < 1; WKY: *F*_(2,42)_ = 1.5, *p* = 0.228, $\eta_{\text{p}}^{2}$ = 0.07). Thus, irrespective of stress, Wistar and WKY rats both increased the size of their licking clusters as the solution consumed increased in concentration. While lick cluster size increased concentration in WKY rats, their overall affective response to each solution appeared greatly reduced relative to Wistars. This confirms the suggestion from the anticipatory contrast tests that, while the hedonic reaction of WKY rats to palatable sucrose is materially blunted, it is not entirely absent.

## Discussion

The present study highlighted how from the perspective of potential stress-diathesis animal modeling of depression that the application of a mild stressor sequence results in qualitatively different effects to those exhibited by WKY rats in tests of anticipatory and consummatory anhedonia. Stress did not significantly exacerbate any behavioral differences exhibited by WKY rats. These results suggest the potential utility of WKY rats in modeling aspects of depressive symptomatology, but a caution for a more nuanced interpretation of the biological factors driving alterations in hedonic behavior. Measures of total consumption should not be considered biologically equivalent to other measures of palatability, and anticipatory and consummatory phases of hedonic responses can be behaviorally and biologically dissociated.

Consumptive behavior during the sucrose preference test has been the most frequently used approach to infer potential impairments in hedonic response in several animal models of depression (e.g., unpredictable chronic mild stress, olfactory bulbectomy, social defeat, as evidenced by Romeas et al., 2009; Burstein and Doron, 2018; Iñiguez et al., 2018; Antoniuk et al., 2019). While the present study indicated the presence of decreased consumptive behavior of sucrose solution in WKY rats, as indexed by the total amount of solution ingested, a potential confound arises from the fact that WKY rats were of lower overall bodyweight than their age-matched Wistar counterparts. When bodyweight is corrected for, the differences between WKY and Wistar rats were removed or greatly reduced. Previous findings have been somewhat mixed here, where for instance marked increases in sucrose consumption have been observed in WKY rats (Papacostas-Quintanilla et al., 2017). Other previous studies have not included a comparator strain as a control (e.g., Tacchi et al., 2008), while others have found increased bodyweight of WKY rats compared to Wistars (Dommett and Rostron, 2013). These discrepancies should be borne in mind when considering the reliability of consumptive measures in this context and overall interpretation of such literature.

Beyond overall consumption, the most general difference observed between WKY and Wistar rats was the far lower levels of lick cluster size seen in the WKY animals when consuming sucrose. Importantly, this difference in the lick cluster size measure of hedonic response is unlikely to be a product of either the differences in the amounts of the solutions consumed—because lick cluster size and consumption vary independently (Davis and Smith, 1992; Spector et al., 1998; Dwyer, 2012), as they do here concerning the effects of stress—or the differences in body weight. We are aware of no reports that bodyweight influences lick cluster size and in the current experiments there was no relationship between weight and lick cluster size^5^. Because lick cluster size is a direct indication of the palatability or hedonic response to the solution being consumed, this measure can be considered a clear analog of consummatory anhedonia as seen in depression (Rizvi et al., 2016; Wu et al., 2017). In the sucrose consumption test involving single solution presentations of different concentrations of sucrose, WKY rats remained somewhat sensitive to the difference between sucrose concentrations, exhibiting larger lick cluster sizes for higher concentrations. Thus, consummatory hedonic responses were not entirely absent in WKY rats, but very markedly blunted. In addition to deficits in consummatory pleasure, anticipatory hedonic responses are also reported to be significantly diminished in depressed patients (Bylsma et al., 2008; Sherdell et al., 2012) and may relate to impairments in episodic future thinking related to hedonic responses (Hallford et al., 2020). Results from the anticipatory contrast test conducted suggested that WKY rats were able to learn the contingencies in force during this study—displaying lower consumption of 4% sucrose in the context where 32% sucrose was anticipated later in the session. This finding is in line with the previously reported findings of sensitivity to positive and negative contrast in WKY rats (Dommett and Rostron, 2013). In the present study, negative anticipatory contrast effects on the lick cluster size measure displayed a different temporal profile to that of contrast effects on consumption and were only reduced for the 4% solution by the end of the contrast training period. While the size of this anticipatory hedonic effect was far smaller than in Wistar controls, mean lick cluster sizes in WKY rats were low overall. There was a clear possibility that floor effects could limit the opportunity for WKY rats to display contrast effects on lick cluster size. Previous evidence is consistent with consumption and hedonic changes being independent effects of contrast, or with the consumption changes being the result of hedonic devaluation (Wright et al., 2013). Such measures may be very important from a translational perspective, as the presentation of anhedonic symptoms has been related to poorer prognosis and treatment sensitivity (e.g., Moos and Cronkite, 1999; McMakin et al., 2012).

The impact of a sequence of stressful events on sucrose solution ingestive behavior was quite distinct to the baseline differences observed in WKY rats. The stressors applied in the present study did result in generally lower levels of consumption across both the anticipatory and consumption phases of the experiment, an effect that survived bodyweight correction. Many prior studies have inferred hedonic deficits from reduction in sucrose consumption and/or preference. This has been particularly common in the case of the chronic mild stress model for depression (e.g., Willner et al., 1987; Papp et al., 1991; Muscat and Willner, 1992; Forbes et al., 1996). The current results reflect prior reports that chronic mild stress can reduce sucrose consumption, but the effects of stress did not extend to the more direct measure of hedonic reactions through the analysis of lick cluster size. That is not to suggest that chronic stress cannot produce hedonic deficits (indeed, see Dwyer, 2012; Clarkson et al., 2018; for reports of just such effects), especially because the current stress protocol was deliberately chosen to be less intense than those typically used. Instead, the present study serves as a reminder that stress effects on consumptive and hedonic behaviors are not always overlapping in animal models, and that other non-hedonic influences may drive changes in consumptive behavior in such contexts.

It is, however, noteworthy that the stress procedures applied in the present study did not exacerbate the hedonic deficits indicated by low lick cluster sizes seen in WKY rats, suggesting that this combination of parameters is not a viable stress-diathesis model. While this finding is consistent with some prior reports (e.g., Paré and Kluczynski, 1997; Nam et al., 2014) possibly the large endogenous deficit seen in the non-stressed WKY animals produced a floor effect which could obscure any additional stress effect. However, this explanation is challenged by the fact that stress effects on consumption *per se* were not so obviously affected by potential floor effects and there was no stress-induced enhancement of the WKY response for consumption. It is interesting to consider that a stressor paradigm that induced a deficit *per se* could not exacerbate a qualitatively distinct deficit in WKY rats. The present results suggest that translation of the complexity of experiences of depression-precipitating stressful events in humans to stress-diathesis models in rats may require considerably more effort to validate (Willner and Belzung, 2015).

Despite the lack of findings of stress-induced exacerbation on sucrose ingestive behavior in WKY rats, there was a strong effect of the repeated exposure to the brief swim test in these animals. The finding of generally greater immobility for WKY rats along with the fact WKY show greater increases in immobility across tests is consistent with previous results (e.g., Paré, 1989a, b; Paré and Redei, 1993; Paré and Kluczynski, 1997; López-Rubalcava and Lucki, 2000; Rittenhouse et al., 2002; Nam et al., 2014). Because most swim test paradigms previously reported in the literature are far longer (e.g., involving a 15 min pre-test and a 5 min test period) than the 2 min sessions used here, the current results are novel in showing significant differences between Wistar and WKY rats despite a significantly reduced swim time. This has an important welfare implication in that reliable WKY deficits, and by implication, other swim test effects can be identified while reducing the overall exposure to the stressful environment of the test. Progressive increases in immobility of WKY rats with repeated exposure to swim tests potentially demonstrate that different behavioral parameters are differentially sensitive to stressors in these animals. However, there are two important limitations to this finding. First, the brief swim test was only administered within the context of a broader chronic mild stress paradigm, and future studies should consider whether such brief swim procedures are sensitive to WKY vs. Control differences in the absence of additional stressors. The second limitation relates to the interpretation of the translational relevance of changes in swim test behavior in the context of depression. Some authors elegantly argue that swim testing in rodents has little construct validity concerning depression symptoms in humans (Commons et al., 2017), but may more accurately index the ability of an animal to cope with stress. From this perspective, it could be speculated that WKY rats did not cope with the chronic mild stress paradigm as well as Wistar rats did, but that this differential sensitivity to stress did not manifest at the level of hedonic responding.

A final very important limitation to acknowledge regarding this study was the focus on male rats, an unfortunate consequence of resource limitation. It has long been proposed that there is a female bias in the incidence of depression (Weissman et al., 1993) and that there are sex-dependent differences in the expression of depressive symptoms (Cavanagh et al., 2017). Differential sex effects can also be observed concerning stress vulnerability (Bangasser and Valentino, 2014; Bangasser and Wicks, 2017; Bangasser and Wiersielis, 2018; Bangasser et al., 2018; Wellman et al., 2018) and thereby the potential ability of stress to modulate ongoing depressive symptoms. In animal studies, sex is an important variable in the expression of sucrose binging-like behavior in WKY rats (Papacostas-Quintanilla et al., 2017). Other work suggests that female WKY rats exhibit less anhedonia than male WKY rats (Burke et al., 2016), although direct non-consumptive measures of anhedonia were not employed in this study. Future work should more thoroughly test the effect of sex of WKY rats on stress-induced exacerbation of hedonic responses.

A greater appreciation of the heterogeneity of depressive symptoms and neurobiological substrates underpinning these effects makes discussion of the “best” animal models of depression somewhat superficial, especially in the context of the hedonic tests applied here, that have been rarely reported in the previous literature. However, understanding the behavioral profiles of animal models with greater resolution will greatly help in consideration of their translational relevance, and which systems and circuits might be involved in the expression of these effects. WKY rats were found to be sensitive to some degree to differences between the present and anticipated sucrose solution in a contrast study, but show greatly attenuated hedonic reactions to them. Thus, WKY animals display a specific hedonic deficit rather than a general insensitivity to reward. Further, a mild stress paradigm did not exacerbate the effects on hedonic reactions in WKY rats. Altogether, the current results are consistent with previous proposals that WKY rats are a promising laboratory model for the study of hedonic aspects of depression (Overstreet, 2012; Nam et al., 2014; Willner and Belzung, 2015; Wang et al., 2017) but further work will be needed to establish a reliable diathesis-stress procedure.

## Data Availability Statement

The raw data supporting the conclusions of this article will be made available by the authors, without undue reservation, to any qualified researcher.

## Ethics Statement

The animal study was reviewed and approved by the Cardiff University Animal Welfare and Ethical Review Board (AWERB) and all experiments were conducted in accordance with the United Kingdom Animals Scientific Procedures Act, 1986.

## Author Contributions

All authors contributed to the overall design of the studies, with RW and DD determining the exact schedule of testing. RW performed the experiments and collated the data for analysis. RW and DD performed the statistical analysis. These experiments were initially prepared for inclusion in her Ph.D. thesis by RW, and the initial draft of the article written by DD and GG. All authors contributed to and approved the final draft of the article.

## Conflict of Interest

RW received a BBSRC CASE studentship cofunded by Eli Lilly & Co., DD has supervised two PhD students cofunded by Eli Lilly & Co., and GG is employed by Eli Lilly & Co. The authors declare that they have no other potential conflicts of interest, financial or otherwise, related to this work.

## Footnotes

^1^The removal of this animal does not impact on the significance (or otherwise) of main effects of strain, stress, or the interaction between them any analysis presented below.

^2^Although the test consumption of sucrose represents only a fraction of the rats overall energy intake, the caloric requirements of animals generally scale such that they relate to weight (Kleiber, 1947). If this scaling is applied to the consumption tests described above, the main effect of rat strain is removed (*F* < 1), but the remaining features of the analysis are unaffected (with the exception of the 4-way block × contrast × strain × stress interaction that becomes significant (*F*_(3.9,169.8)_ = 2.5, *p* = 0.049, $\eta_{\text{p}}^{2}$ = 0.05). Therefore, differences in bodyweight may have contributed to the lower overall consumption exhibited by WKY rats. This, together with the dissociation between stress effects on consumption and lick cluster size measures, reinforces the idea that consumption measures alone can be ambiguous indicators of hedonic responses.

^3^If the bodyweight scaling described previously is applied to the consumption tests reported here the main effect of rat strain is greatly reduced in effect size but remains significant (*F*_(1,44)_ = 212, *p* < 0.001, $\eta_{\text{p}}^{2}$ = 0.33). The remaining features of the analysis are unaffected by normalization. Therefore, differences in bodyweight may have contributed to the lower overall consumption exhibited by WKY rats.

^4^If the bodyweight scaling described previously is applied to the consumption tests reported here the main effect of rat strain is removed (*F* < 1), but the remaining features of the analysis are unaffected. Therefore, differences in bodyweight may have contributed to the lower overall consumption exhibited by WKY rats.

^5^For the negative anticipatory contrast experiment, the correlation closest to being significant was that between weight and lick cluster size for 4% sucrose in the 4–4 condition in Wistar rats, *r*_(24)_ = −0.301, *p* = 0.153. For the consumption experiment, the correlation closest to being significant was that between weight and lick cluster size for 8% sucrose in Wistar rats, *r*_(24)_ = −0.287, *p* = 0.174.
