# Supplementary material for: Wistar Kyoto Rats Display Anhedonia In Consumption but Retain Some Sensitivity to the Anticipation of Palatable Solutions
Source: Front Behav Neurosci. 2020 Jun 3;14:70. doi: 10.3389/fnbeh.2020.00070 (PMC7283460; doi:10.3389/fnbeh.2020.00070)
Supplement: Supplementary file 1 [file Data_Sheet_1.docx]

Supplementary Material – Wright, Gilmour, Dwyer

# Details of social and environmental stressors

Table S1 Social and environmental stressors. Three of the five stressors were randomly applied each week in addition to social isolation and unenriched home cages. Stressor identity and the day that the stressor was given were randomly allocated to minimise habituation to the stress procedure over time.

| *Stressor* | *Duration* | *Description* |
| --- | --- | --- |
| Wet Bedding | < 4 Hours | Rats were transferred to a different cage where the sawdust had been dampened with approximately 300 ml of cold water. |
| Overnight Illumination |  | Light-dark cycle was temporarily reversed. This manipulation was never done on consecutive days. |
| Cage Swap |  | The cages of two rats were randomly swapped including water bottles. Rats remained in the cage of the unfamiliar rat until their cages were next cleaned (maximum of 7 days). |
| Pair-Up | < 12 Hours | Rats were paired at random, within their strain, and left housed together overnight. All pair-ups included a defending male and an intruder male, as one rat was placed into the home cage of another instead of into clean ‘neutral’ home cage. Which rat was to be the intruder/defending male was randomly allocated. * |
| Brief-Swim Test | < 2 Minutes | Rats were carefully lowered into a black container of water, measuring 33.5 cm by 23 cm (H × D). The temperature of the water was maintained at 20-22 º C. To minimize escape, the water surface was kept at 16-17 cm from the lip of the container. Any rat which escaped was placed back in the water for a maximum of four times after which the trial was terminated. As two rats (1 Wistar and 1 WKY) were run simultaneously in separate containers, trials were terminated for both rats. Upon trial termination or once 2-min had elapsed, rats were removed from the water and carefully dried off before being replaced in their home cage. Water was replaced after four rats had been tested. |

*Rats were carefully monitored for signs of fighting. One rat received bite marks after being paired up and so no longer underwent this manipulation.

# Dates of stressor application and experimental procedures

Table S2 Timing of stress and experimental procedures.

| ***Date*** | ***Stress /Husbandry*** | ***Experiment details*** |
| --- | --- | --- |
|  |  |  |
| 17/10 |  | Arrival - rats weight-matched and separated into Stress and No-Stress conditions |
| 18/10 – 23/10 | Adapt to home cage environment |  |
| 24/10 | Cage Swap |  |
| 25/10 | Pair Up |  |
| 26/10 | Overnight Illumination | Food restriction started |
| 27/10 |  |  |
| 28/10 |  | Habituate to test equipment (all) |
| 29/10 |  | Habituate to test equipment (all) |
| 30/10 | Overnight Illumination | Habituate to test equipment (all) |
| 31/10 | **Brief Swim test 1** | Pre-train_NAC |
| 01/11 |  | NAC_1 – session block 1 |
| 02/11 |  | NAC_2 – session block 1 |
| 03/11 | Pair up | NAC_3 – session block 1 |
| 04/11 | Overnight Illumination | NAC_4 – session block 1 |
| 05/11 |  | NAC_5 – session block 2 |
| 06/11 |  | NAC_6 – session block 2 |
| 07/11 | Wet Cage | NAC_7 – session block 2 |
| 08/11 | Wet Cage | NAC_8 – session block 2 |
| 09/11 |  | NAC_9 – session block 3 |
| 10/11 |  | *Rest* |
| 11/11 |  | NAC_10 – session block 3 |
| 12/11 | Pair up | NAC_11 – session block 3 |
| 13/11 |  | NAC_12 – session block 3 |
| 14/11 | Cage Swap | NAC_13 – session block 4 |
| 15/11 |  | NAC_14 – session block 4 |
| 16/11 | **Brief Swim Test 2** | *Rest* |
| 17/11 |  | NAC_15 – session block 4 |
| 18/11 |  | NAC_16 – session block 4 |
| 19/11 | Overnight Illumination | NAC_17 – session block 5 |
| 20/11 |  | NAC_18 – session block 5 |
| 21/11 | **Brief Swim test 3** | NAC_19 – session block 5 |
| 22/11 | Overnight Illumination | NAC_20 – session block 5 |
| 23/11 |  | NAC_21 – session block 6 |
| 24/11 |  | *Rest* |
| 25/11 |  | NAC_22 – session block 6 |
| 26/11 | Cage Swap | NAC_23 – session block 6 |
| 27/11 | Wet Cage | NAC_24 – session block 6 |
| 28/11 | Pair up | NAC_25 – session block 7 |
| 29/11 |  | NAC_26 – session block 7 |
| 30/11 |  | *Rest* |
| 01/12 |  | *Rest* |
| 02/12 | Overnight Illumination | NAC_27 – session block 7 |
| 03/12 |  | NAC_28 – session block 7 |
| 04/12 | Cage Swap | NAC_29 – session block 8 |
| 05/12 |  | NAC_30 – session block 8 |
| 06/12 | **Brief Swim test 4** | NAC_31 – session block 8 |
| 07/12 |  | NAC_32 – session block 8 |
| 08/12 |  |  |
| 09/12 | Pair Up |  |
| 10/12 |  |  |
| 11/12 | Wet cage | Cons_T1 |
| 12/12 | Wet cage | Cons_T2 |
| 13/12 |  | Cons_T3 |
| 14/12 |  |  |
| 15/12 |  |  |
| 16/12 | Pair Up | Cons_T4 |
| 17/12 | Cage Swap | Cons_T5 |
| 18/12 |  | Cons_T6 |
| 19/12 |  |  |
| 20/12 | **Brief swim Test 5** |  |
| 21/12 |  | Cons_T7 |
| 22/12 |  | Cons_T8 |
| 23/12 |  | Cons_T9 |

# Animal weight data and analysis during the Anticipatory Contrast Study

Table S3 Weights and percentage of free-feeding weights of animals during the anticipatory contrast study period. Data is shown as means across the period (with SEM).

|  | Wistar No-Stress | Wistar Stress | WKY No-Stress | WKY Stress |
| --- | --- | --- | --- | --- |
| Mean weight (g) | 260.5 (4.1) | 260.9 (5.8) | 230.3 (3.5) | 223.3 (5.6) |
| Percentage of free-feeding weight | 100.5 (1.1) | 101.6 (0.8) | 101.3 (0.7) | 102.0 (0.9) |

Both the weight (g) and percentage of free-feeding weights were analysed with two-way between subjects ANOVA with factors of strain (WKY v Wistar) and stress (Stress v No-Stress). For the weight data, this revealed a main effect of strain (F(1, 44) = 44.44, p < 0.001, ƞ^2^_p_ = 0.524), and no significant effect of stress (F(1, 44) = 0.46, p = .503, ƞ^2^_p_ = 0.010) or interaction between the two factors (F(1, 44) = 0.58, p = .451, ƞ^2^_p_ = 0.013). For the percentage of free-feeding weight data, this revealed no significant effects of strain (F(1, 44) = 0.50, p = .485, ƞ^2^_p_ = 0.011) or stress (F(1, 44) = 1.034, p = .315, ƞ^2^_p_ = 0.023), nor an interaction between the two factors (F(1, 44) = 0.09, p = .765, ƞ^2^_p_ = 0.002). Thus, although the WKY and Wistar strains differed in overall bodyweight, they were matched in terms of their percentage of free-feeding weights, and there was no effect of stress (on interaction between strain and stress) on either measure.

# Animal weight data and analysis during the Consumption Study

Table S4 Weights and percentage of free-feeding weights of animals during the consumption study period. Data is shown as means across the period (with SEM).

|  | Wistar No-Stress | Wistar Stress | WKY No-Stress | WKY Stress |
| --- | --- | --- | --- | --- |
| Mean weight (g) | 290.4 (5.5) | 287.3 (5.4) | 253.9 (3.8) | 245.9 (4.9) |
| Percentage of free-feeding weight | 112.0 (1.6) | 112.0 (1.2) | 111.8 (1.2) | 112.5 (1.6) |

Both the weight (g) and percentage of free-feeding weights were analysed with two-way between subjects ANOVA with factors of strain (WKY v Wistar) and stress (Stress v No-Stress). For the weight data, this revealed a main effect of strain (F(1, 44) = 61.01, p < 0.001, ƞ^2^_p_ = 0.581), and no significant effect of stress (F(1, 44) = 1.25, p = .270, ƞ^2^_p_ = 0.028) or interaction between the two factors (F(1, 44) = 0.24, p = .630, ƞ^2^_p_ = 0.005). For the percentage of free-feeding weight data, this revealed no significant effects of strain (F(1, 44) = 0.01, p = .924, ƞ^2^_p_ < 0.001) or stress (F(1, 44) = 0.07, p = .788, ƞ^2^_p_ = 0.002), nor an interaction between the two factors (F(1, 44) = 0.06, p = .806, ƞ^2^_p_ = 0.001). Thus, although the WKY and Wistar strains differed in overall bodyweight, they were matched in terms of their percentage of free-feeding weights, and there was no effect of stress (on interaction between strain and stress) on either measure.

.
